# Management of intracardiac thrombosis in newborns: a case series and a narrative review of the literature

**DOI:** 10.3389/fcvm.2025.1659312

**Published:** 2025-08-06

**Authors:** Domenico Umberto De Rose, Federico Mecarini, Cosimo Marco Campanale, Flaminia Pugnaloni, Chiara Passarella, Aurelio Secinaro, Giorgio Bracaglia, Matteo Luciani, Alessandra Toscano, Irma Capolupo, Andrea Dotta

**Affiliations:** ^1^ Neonatal Intensive Care Unit, “Bambino Gesù” Children’s Hospital IRCCS, Rome, Italy; ^2^ Neonatology and Neonatal Intensive Care Unit, Department of Pediatrics, Local Health Authority of Viterbo (ASL Viterbo), Santa Rosa Hospital, Viterbo, Italy; ^3^ Perinatal Cardiology Unit, “Bambino Gesù” Children’s Hospital IRCCS, Rome, Italy; ^4^ Advanced Cardiothoracic Imaging Unit, “Bambino Gesù” Children’s Hospital IRCCS, Rome, Italy; ^5^ Pediatric Hematology and Oncology Unit, “Bambino Gesù” Children’s Hospital IRCCS, Rome, Italy

**Keywords:** heparin, neonate, enoxaparin, cardiac thrombosis, FIRS, echocardiography

## Abstract

**Background:**

Neonatal intracardiac thrombosis (ICT) is an uncommon but increasingly recognized condition that impacts neonatal morbidity and mortality, especially in critically ill term and preterm infants. Management includes supportive care and pharmacological or surgical intervention. This study explores the challenges associated with ICT in neonates.

**Methods:**

We described the clinical presentation and multidisciplinary management of two cases of intracardiac thrombosis. We also reviewed literature from Medline and PubMed using MeSH terms (“intracardiac thrombosis” AND “newborn”).

**Case series:**

In the first case, a very early (day 1) atrial thrombosis was unusually attached at the fossa ovalis and floating between the right and left atrium in an early-term newborn with meconium-aspiration syndrome and fetal inflammatory response syndrome. In the second case, a late-preterm neonate developed a left atrial thrombus after resuscitation at birth, with severe anemia (hemoglobin 5 g/dl) requiring two blood transfusions. In both cases, treatment with low-molecular-weight heparin resolved the thrombus without complications.

**Results:**

Critically ill term and preterm neonates should be carefully monitored due to the increased risk of thromboembolic events. The timing and decision to treat ICTs remain challenging. Supportive therapy is always indicated, including treatment of conditions such as sepsis, dehydration, anemia, and coagulopathy. Anticoagulant therapy with low-molecular-weight heparin (LMWH) offers a favorable risk/benefit ratio, except in neonates at high hemorrhagic risk.

**Conclusion:**

Neonatal intracardiac thrombosis, though rare, requires high clinical suspicion and prompt multidisciplinary management. Early diagnosis and individualized anticoagulant therapy can lead to favorable outcomes while minimizing complications.

## 1 Introduction

Neonatal intracardiac thrombosis (ICT) is a rare but potentially life-threatening condition characterized by the formation of blood clots within the chambers or great vessels of a newborn's heart. Although uncommon, it is becoming more frequently reported, particularly with the expanded use of central venous catheters and advancement in neonatal intensive care. It can have a substantial impact on neonatal morbidity and mortality (1).

The clinical presentation can range from asymptomatic to severe, depending on the thrombus size, location, and potential for pulmonary and systemic embolization, leading to devastating complications such as pulmonary embolism, stroke, or myocardial infarction (2–4).

Early and accurate diagnosis is critical for guiding the management, which typically includes anticoagulation, although invasive interventions may be considered in selected cases (5–10).

Herein, we report two cases successfully treated with anticoagulation, with a brief review of the literature on the incidence and localization of neonatal ICT, pathophysiology, risk factors, and management workflow.

## 2 Methods

In order to review the literature about intracardiac thrombosis in newborns, an extensive literature search in the MEDLINE database (via PubMed) has been performed up to June 10th, 2025. The keywords “intracardiac thrombosis” AND “neonate” OR “newborn” were searched as entry terms as well. Papers written in languages other than English were excluded.

## 3 Case presentation

### 3.1 Patient 1

A female infant weighing 3,670 g was born at 37 weeks of gestational age to a 37-year-old G2P1 Caucasian mother by elective repeat cesarean section. The woman was a heterozygous carrier of the prothrombin G20210A mutation and was managed with enoxaparin. Moreover, pregnancy was complicated by gestational diabetes requiring insulin treatment; vaginal swabs were positive for Escherichia coli. Fetal echocardiography (performed because of maternal diabetes) revealed no anomalies.

At birth, the infant was non-vigorous due to meconium aspiration and required resuscitation using endotracheal intubation and tracheal suctioning. Apgar scores were 5 and 7 at 1 and 5 min, respectively. Umbilical arterial blood analysis at the time of birth showed a pH of 7.16 with a base excess of −11 mmol/L. Neurological evaluation at 60 min showed no signs of encephalopathy on Sarnat staging, and amplitude-integrated electroencephalography (aEEG) revealed a trace within normal limits; therefore, the infant did not undergo therapeutic hypothermia, according to the Italian Society of Neonatology neuroprotective cooling protocol.

She was managed with mechanical ventilation (FiO2 100%), surfactant administration (lung lavage with dilute surfactant and then a further bolus dose), umbilical venous catheterization, parenteral nutrition, and empirical broad-spectrum antibiotics.

A transthoracic echocardiogram (TTE) within 1 h of life revealed a redundant, thin, freely mobile hyperechoic structure that appeared to float between the right and left atrium through the patent foramen ovale.

**Figure 1:**
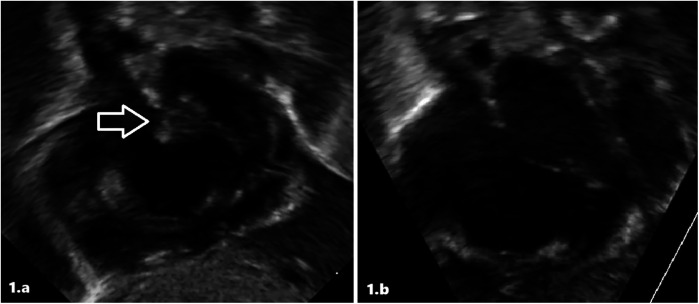
Intracardiac thrombus at diagnosis **(a)** and after treatment **(b)** in patient 1 at echocardiographic scans.

Therefore, she was immediately referred to our III-level Neonatal Intensive Care Unit (NICU). At admission, cardiovascular examination showed normal heart sound, no audible murmur, and no differences between pre- and post-ductal saturation of peripheral oxygen (measured by pulse oximetry) and normal peripheral pulses. TTE demonstrated findings consistent with persistent pulmonary hypertension (PPHN), including a dilated/hypertrophied right ventricle, left ventricular hypertrophy, and a patent ductus arteriosus (PDA) with bidirectional shunting. The diagnosis of the intra-atrial lesion was confirmed; the image was suggestive of a thrombus (Figure 1a). Enoxaparin treatment (150 U/kg twice a day subcutaneously) was started and titrated according to antifactor Xa (anti-Xa) levels monitoring. She needed to be placed on milrinone, vasopressors (vasopressin and norepinephrine), and high-frequency oscillatory ventilation (HFOV), with gradual improvement. We ruled out sepsis and viral infections.

The atrial thrombus was monitored daily with serial ultrasound evaluations. After 5 days of low-molecular-weight heparin (LMWH) treatment, echocardiography showed the resolution of the thrombosis (Figure 1b). Furthermore, the infant underwent a whole-body computed tomography (CT) that revealed no signs of embolism. At resolution of the thrombosis, continuous enoxaparin infusion was initially switched up to two subcutaneous infusions/day, then once/day. The infant was subsequently weaned from ventilator support, with successful extubation performed at 10 days; head magnetic resonance imaging (MRI) revealed no pathologic anomalies. The observation of ICT led to further tests being performed, and also the infant was confirmed as a heterozygous carrier of the prothrombin G20210A mutation. Enoxaparin was discontinued after 23 days of treatment. She was discharged at 34 days of life without any treatment.

At 24 months of life, she is currently receiving psychomotor and speech therapy with five sessions per week. No concerns have been reported regarding sleep, feeding, or social interaction. At the time of the psychological evaluation, Anita engaged positively in the relationship and showed interest in the proposed play materials. Independent walking remains unsteady. Expressive language is characterized by approximately 10 clearly articulated words, which she uses to express her needs. On the Griffiths Developmental Scales, her equivalent age (EA) was 20 months compared to a chronological age (CA) of 24 months (Locomotor Area: EA 18.5 months; Personal-Social Area: EA 20.5 months; Language and Cognitive Area: EA 20.5 months; Eye-Hand Coordination Area: EA 19 months; Performance/Non-verbal Abilities Area: EA 20.5 months).

### 3.2 Patient 2

A 34-year-old secundigravida was admitted to the Obstetric Emergency Department due to decreased fetal movement. Cardiotocography revealed a non-reassuring fetal heart rate pattern and suspected fetal distress. Therefore, an emergency cesarean section was performed at 36 weeks of gestation. The obstetric history was negative for infectious diseases of the TORCH group. The blood group was A RhD positive, and the red blood cell (RBC) antibody screen was negative. Fetal membranes were intact, and vaginal-rectal swabs were negative 6 days prior to delivery. Low-dose aspirin until 34 weeks of gestation and subsequently LMWH were prescribed due to a previous pregnancy that ended in stillbirth at 41 weeks with severe intrauterine growth restriction (IUGR). Antenatal corticosteroids were administered two weeks prior to delivery due to threatened preterm labor.

At birth, the female neonate was born apneic, hypotonic, bradycardic (>60 bpm), and pale and required non-invasive ventilation support. Apgar score was 4 at 1st minute and 8 at 5 min. Birth weight was 2.258 kg (9th percentile). Due to persistent respiratory distress, the newborn was managed with nasal continuous positive airway pressure (nCPAP). No birth defects or abnormal features were found. She received routine vitamin K intramuscularly to prevent bleeding.

A blood gas analysis revealed severe anemia, confirmed in the laboratory, with a haemoglobin of 5 g/dl and a haematocrit of 15.2%. An umbilical venous catheter (UVC) was immediately placed, and a red blood cell transfusion (20 ml/kg) was started at 4 h of life. An echocardiogram revealed PPHN and a PDA with a bidirectional shunt.

Respiratory support was stopped on day 2. Another transfusion was needed due to low haemoglobin (9 g/dl). TTE showed a closed ductus arteriosus, mild tricuspid insufficiency, and moderate mitral insufficiency. Blood counts gradually improved, except for slightly low platelets (90.000 platelets/mm^3^). On the 5th day of life, platelets rose (118.000 platelets/mm^3^) and blood values remained stable (haemoglobin 14.1 g/dl and haematocrit 44.4%). On the 6th day of life, the central line and antibiotics were stopped after infection was definitively ruled out.

**Figure 2:**
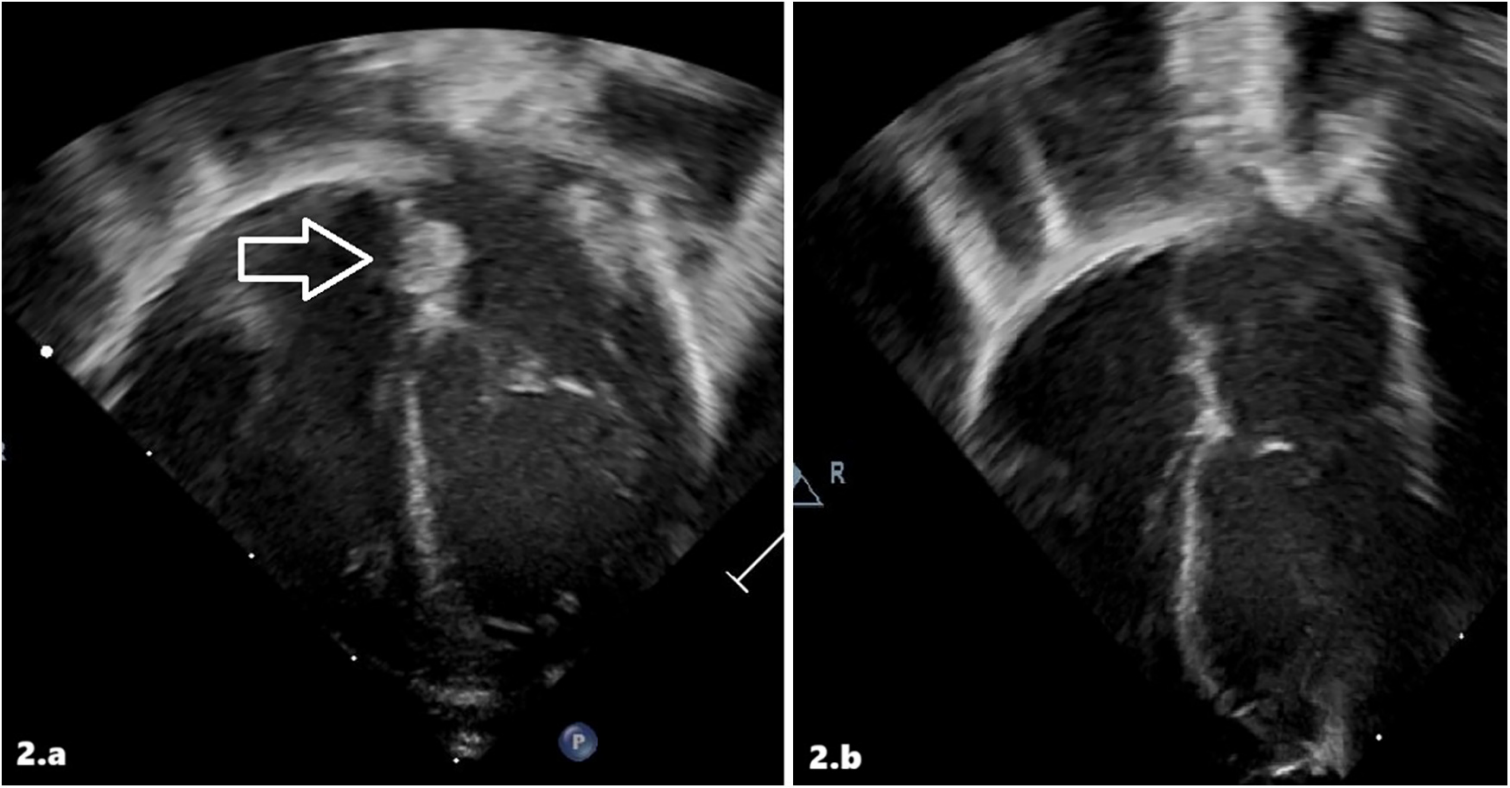
Intracardiac thrombus at diagnosis **(a)** and after treatment **(b)** in patient 2 at echocardiographic scans.

On the 7th day of life, cardiac ultrasound detected a mobile, pedunculated mass in the left atrium attached to the left atrial septum with a maximum diameter of 0.37 × 0.65 cm (Figure 2a). Findings were compatible with the diagnosis of atrial thrombosis, and the newborn was transferred to our III-level NICU. Upon admission to the other hospital, the echocardiographic diagnosis was confirmed. Anticoagulant therapy with LMWH (enoxaparin 170 U/kg twice a day subcutaneously) was started with hematologic monitoring of anti-Xa levels. Abdominal ultrasound was normal. Cerebral ultrasound showed areas of mildly increased echogenicity in deep periventricular white matter, but no evidence of thrombosis. Thrombophilia screening revealed a homozygous mutation in the MTHFR gene (C677 T variant), with normal homocysteine levels. Haematological workup included protein C, protein S, and antithrombin III. On 12th DOL, cardiac angio-CT and echocardiography showed no evidence of intracavitary cardiac mass (Figure 2b). On the 16th day of life, the infant was discharged home with enoxaparin only once/day and then discontinued after a month. We have no data about long-term follow-up.

## 4 Discussion

In this report, we described two cases of intracardiac thrombosis in neonates. To the best of our knowledge, a very early (day 1) atrial thrombosis has not been previously reported in neonates. Given its rapid formation, within a few hours of life, we hypothesized that clotting started during fetal life rather than being catheter-related in the first case we described. The robust fetal inflammatory response syndrome (FIRS) that meconium-stained amniotic fluid (MSAF) could have induced might have led to the clot formation, even a few hours before delivery. Conversely, in the second case, the newborn was born prematurely, and a UVC was immediately placed because of respiratory distress, and the thrombosis could be related to the central line.

### 4.1 Incidence and localization

Thromboembolic events (TEs) are uncommon during childhood. The incidence of symptomatic TEs in children is 0.07/10,000 compared to 2.5%–5% of adults (11–14). Newborns are at the greatest risk, with an overall incidence of 2.4 per 1,000 admissions to the NICU (11, 13). In the last decades, the incidence of TEs has increased due to a higher detection rate and increasingly invasive therapeutic interventions, including the use of vascular catheters such as the umbilical venous catheter (UVC), the umbilical arterial catheter (UAC), and the central venous catheter (CVC). Nowadays, 15% of all newborns are admitted to the NICU, and a UVC is placed in more than 50% of preterm neonates with a birth weight of less than 1,000 g (13, 15). It is estimated that the incidence of catheter-associated symptomatic thrombosis is approximately 1%, while asymptomatic thrombosis is 20%–30% (16).

Furthermore, TEs can be both venous and arterial. The most common sites of arterial thrombosis are renal artery thrombosis, arterial vascular catheter access sites, and cerebral arteries (arterial ischemic stroke) (1). Venous thromboses are mainly located in the renal vein, portal vein, inferior and superior vena cava, femoral and axillary veins, cerebral sinovenous tract, and right atrium (1, 17, 18).

Intracardiac thrombosis (ICT) is a rare and life-threatening condition: it usually occurs when there is low flow, which results in blood stasis. Right atrial thrombosis is the most common intracardiac form in newborns and is a well-known complication related to CVC placement (14, 19). Symptomatic intracardiac thrombosis can present as a new murmur or heart failure, in addition to the catheter malfunction (20). In addition, isolated cases of thrombotic lesions of the tricuspid valve and thrombosis of the left atrial appendage have been reported in healthy infants. A thrombus on the left side of the heart is more typical of congenital heart disorders (CHDs), even if reported in newborns with coagulation disorders or as paradoxical emboli in neonates with patent foramen ovale (PFO) (21, 22).

### 4.2 Pathophysiology

The pathogenesis of neonatal thrombosis is complex and multifactorial. In 1,845, Virchow postulated that three factors were critical in the development of thrombosis: impairment of blood flow (stasis), vascular injury, and alterations of the coagulation (hypercoagulability). The Virchow triad is still valid in addressing the etiology of thrombosis in neonates (13).

Newborns have physiological differences in the hemostatic system compared to children and adults. The hemostatic system has a dynamic development during infancy and childhood (23).

Knowledge of the development of hemostasis is essential for the prevention, diagnosis, and treatment of thrombosis in a critically ill neonate. Neonatal concentrations of most clotting factors, turnover rates, and the ability to regulate thrombin and plasmin differ profoundly from adults (24).

On the pro-coagulant side, vitamin K-dependent coagulation factors (factors II, VII, IX, X) and components of the contact system (factor XI, factor XII, prekallikrein, and high molecular weight kininogen) are reduced in the neonatal period. In contrast, factors VIII and von Willebrand are elevated. Coagulation inhibitors (protein C, protein S, antithrombin, heparin cofactor II) have a low concentration in plasma, counterbalancing the reduced clotting potential of neonatal plasma (24, 25).

Neonatal fibrinolytic activity is reduced due to both decreased plasma activity of plasminogen and increased plasma levels of plasminogen activator inhibitor (PAI). Neonatal platelets have been reported to be hypo-reactive. This deficiency seems to be balanced by increased von Willebrand factor activity, resulting in overall normal platelet function. Consequently, healthy newborns may produce a lower clot consistency due to impaired platelet function and a physiological deficiency of coagulation factors (1, 17, 24).

The neonatal hemostatic system is balanced, neither promoting hemorrhage nor thrombosis, by the protective effects of the physiological deficiencies of coagulation inhibitors, as well as by the reduced fibrinolytic capacity. On the other hand, it appears more easily compromised by external factors due to the poor reserve capacity. Furthermore, neonates are born with a high hematocrit and tend to contract their intravascular volume within the first days of life, making them even more prone to thromboembolic events. Thus, some categories of neonates, such as preterm or sick newborns, are extremely vulnerable and predisposed to hemorrhagic or thrombotic complications (13, 26, 27).

### 4.3 Maternal and risk factors

Different triggering risk factors may contribute to the development of TEs in neonates. They can be divided into two main categories: maternal or neonatal (Table 1) (11, 13, 28, 29).

**Table 1:** Risk Factors for Neonatal Thromboembolic Events (TEs).

Category		Risk factors
Maternal		- Infections - Placental diseases - Diabetes mellitus - Hypertension - Preeclampsia - Dyslipidemia - Metabolic syndrome - Antiphospholipid syndrome - Inherited thrombophilia - Premature rupture of membranes
Neonatal	Congenital	- Perinatal asphyxia - Prematurity - Low birth weight - Congenital heart diseases - Arrhythmias - Congenital prothrombotic disorders - Metabolic disorders - Renal diseases
	Acquired	- Intravascular catheterization - Sepsis - Dehydration - Shock - Inflammatory conditions - Mechanical ventilation - Antibiotic therapy - Parenteral nutrition - Blood transfusion - Major surgery - Liver dysfunction

Anyway, the most important risk factor for the development of thrombosis during the neonatal period is the presence of catheterization (30). Consequently, the vessels most involved are those frequently used for catheterization. Together with catheterization, asphyxia, sepsis, and prematurity are among the most important risk factors for neonatal thrombosis (17). Preterm infants have a high risk of TEs because of the combination of high prothrombotic activity, low levels of natural anticoagulants, and various imbalances in the fibrinolytic systems (31).

Congenital heart diseases (CHDs) significantly increase the risk of TEs. In infants younger than 6 months of age with CHD, the estimated incidence of venous TE is about 50% and even higher in arterial TE (70%) (32). Cyanotic CHDs cause secondary polycythemia, resulting in hyperviscosity, reduced blood flow, and a high risk of thrombosis. Additionally, patients with CHDs may undergo surgical procedures, trans-catheter interventions, prolonged central line use, intensive blood product use, post-extracorporeal membrane oxygenation, and heart transplantation. These conditions increase the risk of TE events in the neonatal period (1, 33). In complex CHDs with the possibility of right-to-left shunt, paradoxical embolism may also occur (34).

Newborns are particularly susceptible to thrombo-embolic complications, and acquired predisposing factors could increase their susceptibility. Of these, meconium aspiration syndrome (MAS) is a leading cause of morbidity and mortality in term infants. MSAF occurs in about one of every seven pregnancies, but only 5% of newborns exposed to MSAF develop MAS and severe respiratory distress, often requiring mechanical ventilation (35). Recently, different authors have reported that MAS could lead not only to severe lung damage, but also to a well-defined FIRS (35, 36). FIRS is a systemic inflammatory condition affecting the fetus, characterized by elevated inflammatory mediators, particularly interleukin-6 (IL-6), in the fetal circulation, typically triggered by intra-amniotic infection, inflammation, or hypoxic stress; it's often considered a preclinical stage of neonatal sepsis and a marker of fetal distress due to infection or sterile inflammation (37). The key diagnostic marker for Fetal Inflammatory Response Syndrome (FIRS) is an elevated concentration of interleukin-6 (IL-6) in fetal plasma, typically above 11 pg/ml. Histological evidence of FIRS includes the presence of funisitis and chorionic vasculitis in the placenta (38, 39). The FIRS is associated with a higher incidence of adverse neonatal outcomes and is a risk factor for severe neonatal morbidity or death, according to data from a meta-analysis (40). The spectrum of adverse neonatal outcomes due to FIRS includes an increased risk of early-onset neonatal sepsis with elevated inflammatory markers such as IL-6 and CRP; central nervous system complications like periventricular leukomalacia, intraventricular hemorrhage, and long-term neurodevelopmental delays; respiratory issues including meconium aspiration syndrome, bronchopulmonary dysplasia, and persistent pulmonary hypertension; cardiovascular instability and possible myocardial inflammation; hematologic abnormalities such as thrombocytopenia, coagulopathy, and thrombosis; and gastrointestinal risks like necrotizing enterocolitis, all contributing to higher rates of preterm birth, intrauterine growth restriction, NICU admission, and prolonged hospitalization (37, 40, 41).

In particular, concerning MAS, the exposure to MSAF can lead to airway obstruction, surfactant dysfunction, inflammation, and pulmonary hypertension; FIRS may play a crucial role in sensitizing the fetal lungs to injury, amplifying the inflammatory response (37). MAS associated with perinatal asphyxia could disturb the balance between coagulation and fibrinolysis, leading to a prothrombotic state, as in our first case (42). Indeed, among the pro-inflammatory cytokines, IL-6 has been associated with MAS (43): the ability of IL-6 to mediate platelet responses, including thrombocytosis, platelet hyper-reactivity, and accelerated thrombus formation, has been demonstrated (44).

### 4.4 Hematological prothrombotic risk factors

Inherited thrombophilia is a well-established risk factor for TEs in adults and also increases the risk of thrombosis in neonates, infants, and children (13).

Several authors have reported a high prevalence of hereditary thrombophilia in newborns with renal, portal, or hepatic vein thrombosis or neonatal stroke (1).

Congenital prothrombotic risk factors include deficiency of inhibitors (Antithrombin III, Protein C, Protein S), increased levels of clotting factors (fibrinogen, factor VII, factor VIII, etc.), increased and/or hyperactive platelets, defective fibrinolysis, increased blood and/or plasma viscosity (hypo- or dysplasminogenemia), presence of prothrombotic molecular mutation (Factor V Leiden, Prothrombin G20210A, homozygous mutations of the methylenetetrahydrofolate reductase-MTHFR C677 T and A1298C) (11, 30, 45). Patients with prothrombotic mutations and polymorphisms are more likely to develop thrombosis due to triggering factors, such as catheter placement, prolonged immobilization, or surgery (1). It is estimated that a hereditary prothrombotic factor is present in approximately 50% of children with TEs (46). The most frequently observed hereditary risk factor was the MTHFR 677C-T mutation, and the second most common was the mutation in factor V Leiden (46).

### 4.5 Imaging

Transthoracic echocardiography (TTE) is generally the first-line imaging modality for diagnosing intracardiac thrombosis in neonates, because it's non-invasive, readily available, and provides real-time imaging of cardiac structures, blood flow, and the presence and mobility of thrombi. Thrombotic lesions in neonates can be particularly challenging to assess, as some may present in an insidious manner. At first evaluation, they can appear smaller than their actual size. A comprehensive echocardiographic evaluation is essential to accurately characterize their nature, shape, site of attachment, dimensions, mobility, and relationship to adjacent cardiac structures. This requires the use of multiple imaging planes, including orthogonal and non-standard (“off-axis”) views. Off-axis imaging may be necessary to visualize lesions that are not readily apparent on conventional echocardiographic views, thereby improving diagnostic accuracy (2, 3).

Chest computed tomography (CT), particularly CT angiography (CTA), can be a valuable tool in the management of intracardiac thrombosis in neonates, providing more detailed anatomical information, especially for complex cases or when the thrombus extends into extracardiac vessels (like pulmonary arteries or aorta) or where TTE is inconclusive or limited (e.g., due to poor acoustic window). This is particularly useful for surgical planning. However, radiation exposure, the need for sedation for CT scans, and the need for intravenous contrast administration require careful consideration (47–49).

### 4.6 Clinical management

Acute management of intracardiac thrombosis includes various approaches: observation and monitoring, pharmacological treatment, or surgery. Each of these therapies can be burdened by side effects, particularly bleeding. Therefore, treatment decisions must be highly individualized and require careful evaluation of both potential benefits and risks, in a tertiary referral center with experienced neonatologists, pediatric cardiologists, and pediatric hematologists (18).

Before starting therapeutic anticoagulation, a complete laboratory work-up is mandatory, including prothrombin time (PT) and activated partial thromboplastin time (aPTT), fibrinogen, complete blood count with platelets (at least >50,000/μl), kidney function tests, and liver transaminases. Thrombophilia screening should also be considered, or at least the evaluation of protein C, protein S, antithrombin III, Activated Protein C resistance, and, if necessary, homocysteine and D-dimer levels.

When right atrium thrombi are associated with CVCs, the catheter should be removed if possible.

The treatment of neonatal ICT is usually anticoagulation (typically with LMWH), except in cases where its use is contraindicated and the risk of bleeding is too high. Data from 31 studies, encompassing 1,063 neonates receiving LMWHs, indicate that major bleeding events were observed in 4.1% of cases (44 neonates). Of these, 13% were intracranial hemorrhages, making it the most common bleeding location (50). Thus, brain ultrasound should be regularly performed before and after treatment, especially in those who are at increased risk of developing an intracranial hemorrhage, such as preterm infants. To achieve a therapeutic target range, a high dose could be required, and therefore, careful titration should be performed, adjusting the next dose according to the anti-factor Xa level four to six hours after the dose (50).

Direct oral anticoagulants are not generally used as a first-line agent in neonates, due to scarce safety and efficacy data, especially in the preterm population (51).

Thrombi with low-risk characteristics [small size (<2 cm) and not pedunculated, mobile, or snake-shaped] can be treated conservatively without anticoagulation, according to a systematic review that included data from infants and children (mean age 3.6 years) with RA thrombi (52). However, age is a key factor (53), and in neonates, smaller thrombi may be considered equally as high-risk (52).

If the intracardiac thrombus is compromising cardiac function, thrombolytic therapy (recombinant tissue-type plasminogen activator - tPA -) could be considered, but its use in neonatal age is limited by the high risk of major bleeding compared to other anticoagulants (54).

Surgical thrombectomy is rarely used in neonatal age, when conventional pharmacological treatment has failed or if the thrombus causes a life-threatening obstruction and urgent resolution is needed due to hemodynamic compromise or rapidly worsening condition. Some specific situations could be the acute myocardial infarction of unknown etiology due to complete occlusion of a coronary artery, life-threatening intracardiac thrombosis immediately after complex cardiac surgery, or aortic arch thrombosis, or superior vena cava syndrome (5–9).

Sometimes surgery is performed under suspicion of a congenital heart disease, but a thrombectomy is then carried out when an occlusion, rather than a congenital defect, is documented. For instance, Omeje et al. described a case of occlusive aortic arch thrombus in a neonate who presented with signs of critical coarctation and successfully underwent surgical thrombectomy on deep hypothermic circulatory arrest (7). Similarly, Williams et al. reported the cases of 2 newborns with coarctation and extensive thrombosis within the aortic arch, initially interpreted as arch obstruction secondary to coarctation, where the obstruction was mechanical and was successfully removed by surgery (8).

The interesting approach of transcatheter mechanical thrombectomy has been recently reported by Herron et al., who removed an occlusive aortic thrombus using an Amplatzer Piccolo™ PDA occluder (Abbott, North Chicago, IL, USA) in a preterm newborn. The procedure was successful with no subsequent distal thromboembolic events (10).

Therefore, multidisciplinary evaluation is essential in determining the best approach on a case-by-case basis, especially given the delicate balance between efficacy and safety in neonatal patients.

## 5 Conclusion

Intracardiac thrombosis is a rare event in newborns that can be correlated to several prenatal and postnatal risk factors (such as fetal inflammatory response syndrome, infections, prematurity, perinatal asphyxia, congenital heart disorders, inherited thrombophilia, metabolic disorders, and the presence of a venous or arterial catheter) (2, 3, 13, 17–19, 26). This thromboembolic event is increasingly reported and can be a potentially life-threatening condition for the newborn. Prompt recognition and treatment are crucial to avoid major complications: the management of newborns with ICTs must be multidisciplinary, with experienced neonatologists, pediatric cardiologists, pediatric hematologists, and pediatric cardiac surgeons.
